# Complete septate uterus with obstructed hemivagina and ipsilateral renal agenesis (OHVIRA) in a young woman—a rare variant of Herlyn–Werner–Wunderlich syndrome

**DOI:** 10.1259/bjrcr.20150241

**Published:** 2016-02-25

**Authors:** Mukesh Surya, Sita Thakur, Kamal Singh, Pawan Soni, Dinesh Sood, Preeti Takkar Kapila

**Affiliations:** ^1^ Department of Radiodiagnosis, DRPGMC Kangra at Tanda, Kangra, India; ^2^ Department of Obstetrics & Gynaecology, DRPGMC Kangra at Tanda, Kangra, India

## Abstract

Obstructed hemivagina and ipsilateral renal agenesis (OHVIRA) is an unusual and special type of Müllerian duct anomaly. Patients usually present in adolescence, soon after menarche, with pain and pelvic mass and rarely with infertility in adulthood. Majority of the cases of OHVIRA have been reported in association with uterus didelphys and the presentation of uterus didelphys with OHVIRA is known as Herlyn–Werner–Wunderlich syndrome. A complete septate uterus with OHVIRA is exceedingly unusual. Less than 30 cases of complete septate uterus with OHVIRA have been reported to date, to the best of our knowledge. We present a rare case of incidentally detected complete septate uterus with OHVIRA in a young woman who presented with acute pain in the right iliac fossa owing to acute appendicitis.

## Summary

Obstructed hemivagina and ipsilateral renal agenesis (OHVIRA) is an unusual and special type of Müllerian duct anomaly (MDA). Patients usually present in adolescence, soon after menarche, with pain and pelvic mass and rarely with infertility in adulthood. Majority of the cases of OHVIRA have been reported in association with uterus didelphys and the presentation of uterus didelphys with OHVIRA is known as Herlyn–Werner–Wonderlich (HWW) syndrome. A complete septate uterus with OHVIRA is exceedingly unusual. Less than 30 cases of complete septate uterus with OHVIRA have been reported to date, to the best of our knowledge. We present a rare case of incidentally detected complete septate uterus with OHVIRA in a young woman who presented with acute pain in the right iliac fossa (RIF) owing to acute appendicitis.

## Clinical presentation

A 22-year-old female presented to the gynaecology department with acute onset of severe pain in the right lower abdomen. The pain was colicky in nature, with associated nausea and vomiting. She attained menarche at the age of 14 years and had a history of mild dysmenorrhoea. She had been married for the last 8 months and cohabited with her husband for the same duration. There was no previous history of an abortion. On physical examination, her pulse rate, blood pressure and respiratory rate were within normal limits. Tenderness was noted in the RIF on deep palpation. On per-vaginal examination, the uterus was anteverted and normal in size. A 6 × 6 cm mass was palpable in the right lower adenexa, which was non-tender and cystic in consistency. This mass was causing a smooth bulge in the right vaginal wall.

## Differential diagnosis

Clinical differential diagnoses were acute appendicitis, twisted right ovarian cystic mass and ruptured tubal pregnancy.

## Investigations and imaging findings

All the biochemical investigations were within normal limits. Urine pregnancy test was negative.

She was referred to the radiology department for ultrasonography (USG). Both transabdominal and transvaginal sonography was performed. 3 MHz curved and 10 MHz linear probes were used for transabdominal sonography, whereas transvaginal sonography was performed with a 6 MHz endocavitory probe.

On sonography, a tubular, non-compressible, blind-ending structure was seen in the RIF measuring 8 mm in diameter, consistent with appendicitis. A small amount of periappendiceal fluid was also noted. The uterus was normal in size, having two endometrial cavities extending up to the external os. There was evidence of a cystic mass with internal echoes measuring approximately 5 × 4 cm in size on the right side of the vagina and communicating with the right endometrial cavity. No fluid was seen in the endometrial cavity on either side. Both ovaries were normal in size and outline. The right kidney was not visualized in the right lumbar region or the pelvis. The left kidney was enlarged in size, measuring 13.4 × 5.6 cm in size.

**Figure 1.**
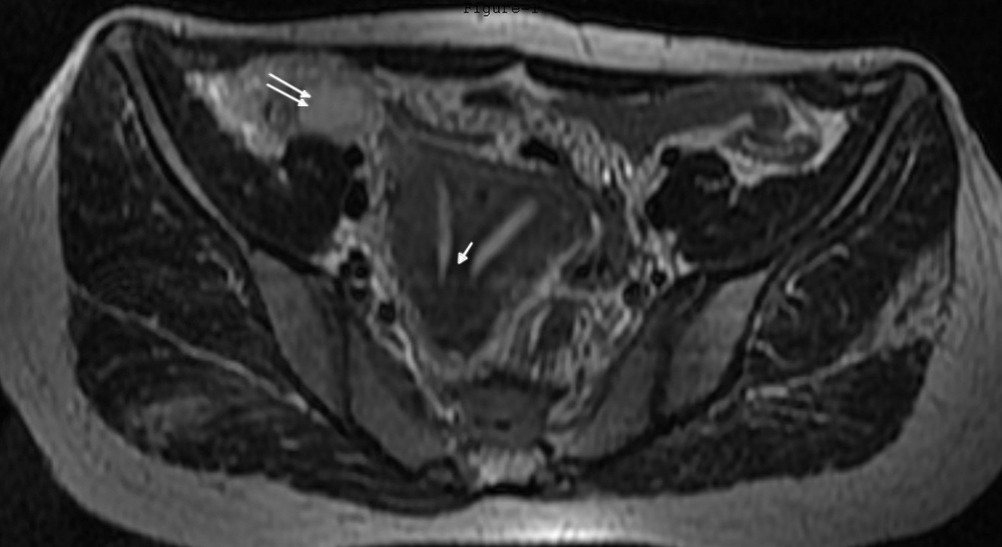
Fast spin echo *T*_2_ weighted (TR 5960 ms, TE 106 ms) axial MRI showing two endometrial cavities separated by a septum, isointense to the myometrium (arrow). Fluid is seen in the periappendiceal region in the right iliac fossa (double arrow).

**Figure 2.**
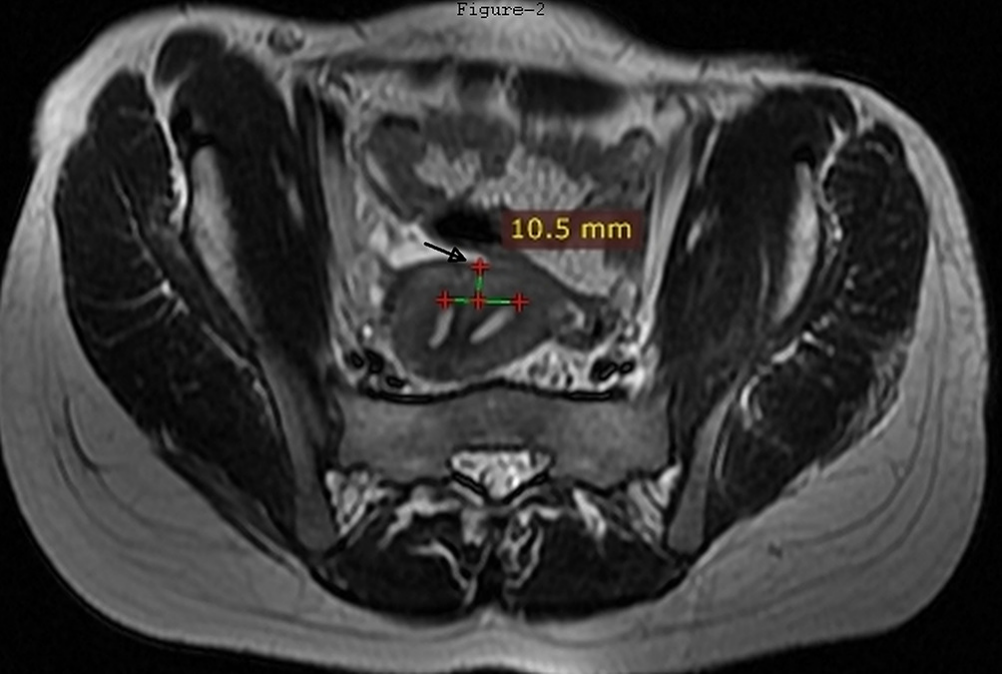
Fast spin echo *T*_2_ weighted (TR 5260 ms, TE 108.2 ms) oblique coronal MRI (parallel to uterus) showing convex fundal contour (arrow). Distance between the fundal apex and the line joining the ostia is 10.5 mm.

**Figure 3.**
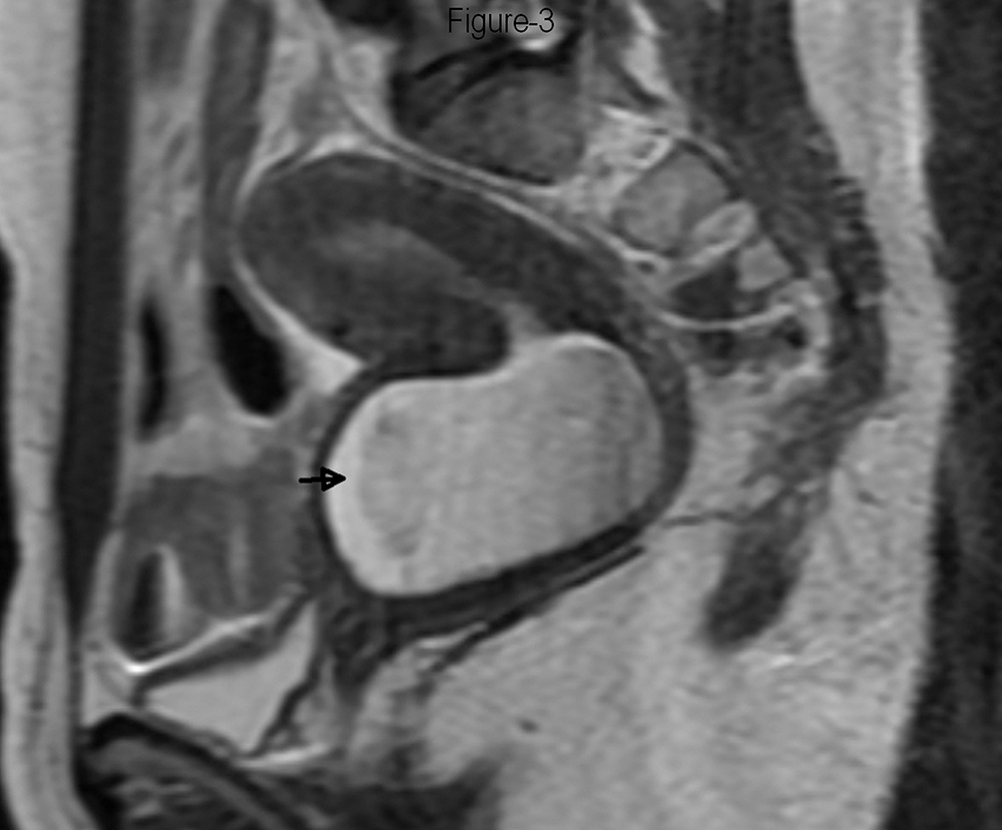
Fast spin echo *T*_2_ weighted (TR 3840 ms, TE 62.8 ms) sagittal MRI showing obstructed right hemivagina containing hyperintense fluid (arrow), communicating with the right endometrial cavity.

**Figure 4.**
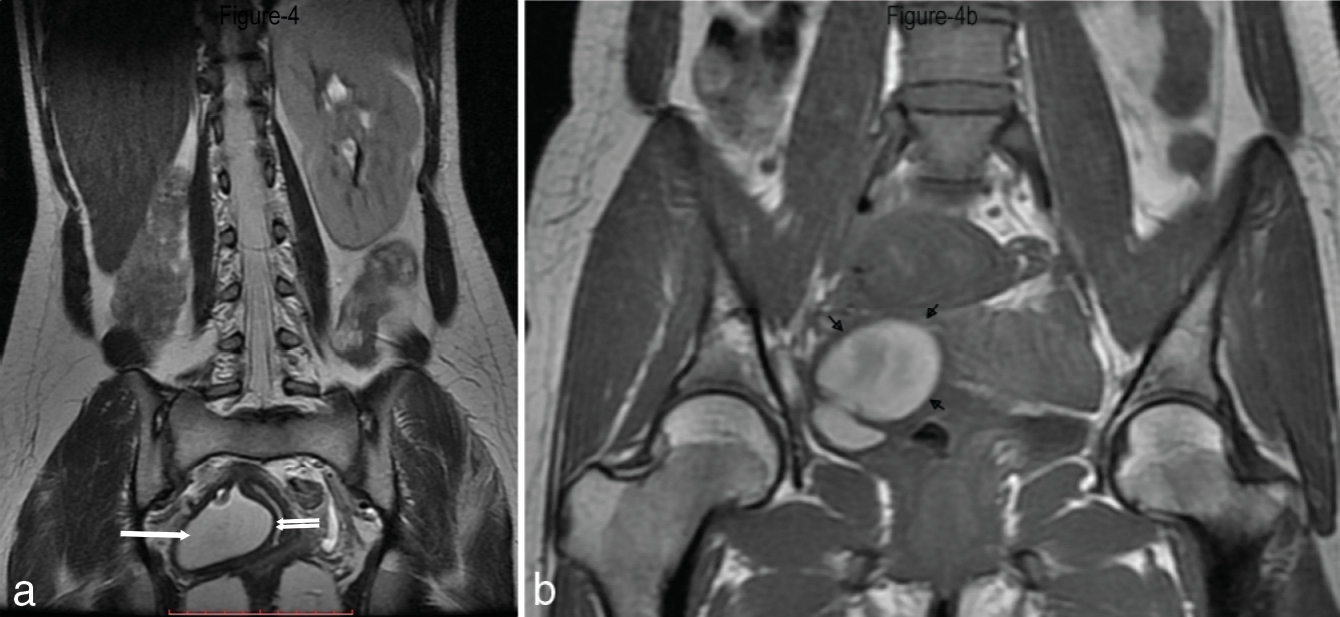
(a) FSE *T*_2_ weighted (TR 5660 ms, TE 61.5 ms) coronal MRI showing the absent right kidney and compensatory hypertrophy of the left kidney. Two hemivaginas are also seen in the pelvis, separated by the septum. The right hemivagina shows evidence of haematocolpos (arrow), while the left hemivagina is normal (double arrow). (b) FSE *T*_1_ weighted (TR 560 ms, TE 7.9 ms) coronal MRI showing the distended right hemivagina filled with hyperintense contents (arrows). FSE, fast spin echo.

An MRI of the pelvic organs was performed to confirm the USG findings. The MRI was performed on a 1.5 T GE Signa Excite (General Electronics Co., Milwaukee, WI). The following pulse sequences were employed: fast spin echo (FSE) *T*_2_ weighted [repetition time (TR) 3840 ms, echo time (TE) 62.8 ms] sagittal; FS-FSE *T*_2_ weighted (TR 5360 ms, TE 62.8 ms) sagittal; FSE *T*_2_ weighted FS (TR 4920 ms, TE 62.6 ms) coronal; FSE *T*_2_ weighted (TR 5660 ms, TE 61.5 ms) coronal, including abdomen for the kidneys; FSE *T*_1_ weighted (TR 560 ms, TE 7.9 ms) coronal; FSE *T*_2_ weighted (TR 5960 ms, TE 106 ms) axial; short tau inversion-recovery (TR 5760 ms, TE 53 ms axial, TI 150  ms) axial; FSE *T*_1_ weighted (TR 440 ms, TE 9.1 ms) axial; FSE *T*_2_ weighted (TR 5260 ms, TE 108.2 ms) oblique coronal *T*_2_ parallel to uterus sequences. Intravenous contrast agent was not used as it was not expected to yield additional information. The MRI revealed two endometrial cavities extending up to the external os. The endometrial cavities were separated by a septum showing intermediate signal intensity, isointense to the myometrium (Figure 1). The uterine horns were not widely separated and on the oblique coronal scans, the distance between the fundal apex and the line between the two ostia was 10.5 mm (Figure 2). The angle between the two endometrial cavities measured 42°. No fluid or blood was observed in the endometrial cavities. A vaginal septum was noted, with the right hemivagina containing 5.5 × 4.5 cm-sized *T*_1_ and *T*_2_ hyperintense contents suggestive of subacute haemorrhagic fluid (Figures 3 and 4a,b). Both ovaries were normal. The right kidney was not visualized on the right side of the abdomen or the pelvis and there was compensatory enlargement of the left kidney (Figure 4a). The appendix measured 7 mm in the transverse diameter, with evidence of periappendiceal fluid (Figure 1).

**Figure 5.**
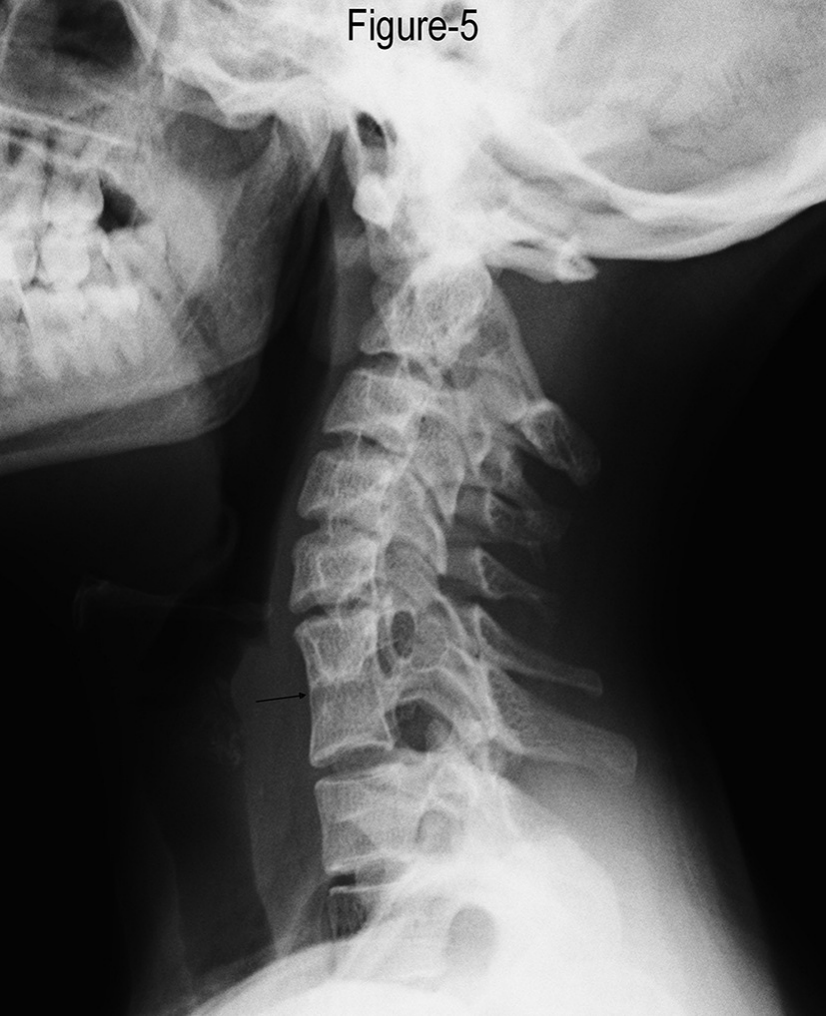
Lateral radiograph showing the fusion of bodies and posterior elements of the sixth and seventh cervical vertebrae (arrow).

When enquired about the history of backache or neck pain, the patient complained of occasional neck stiffness. A lateral radiograph of the cervical spine was performed during her stay in the hospital, suspecting an associated vertebral anomaly, which revealed a block vertebra involving the C6–C7 vertebrae (Figure 5).

Based on the clinical and radiological findings, a diagnosis of complete septate uterus and OHVIRA with acute appendicitis with block vertebra C6–C7 was made.

## Discussion

A failure of fusion of the Müllerian ducts or resorption of the uterovaginal septum between 6 and 12 weeks of intrauterine life leads to a complex spectrum of MDAs. The reported prevalence of MDAs is 1–5% in the general public and 13–25% in females with recurrent abortions. Buttram and Gibbons^1^ proposed an MDA classification in 1979, which was subsequently modified by the American Society for Reproductive Medicine in 1988.^2^ Uterus didelphys constitutes a Type III anomaly, which is a common MDA. However, uterus didelphys with vaginal septum leading to obstruction of menstrual blood with ipsilateral renal agenesis is a special and unusual malformation known as HWW syndrome. A septate uterus has been classified as Type V MDA and is the most common form of MDA, accounting for 55% cases. The septum can be partial or complete, extending up to the external os. The septum may rarely extend into the vagina. In our case, the septum was extending into the vagina with obstructed right hemivagina. Although the majority of the patients with OHVIRA have uterus didelphys, obstructed hemivagina may also be seen with other MDAs such as a bicornuate and septate uterus. Very few cases of complete septate uterus with OHVIRA have been reported to date. A complete septate uterus with OHVIRA was seen in our case. We could trace fewer than 30 cases of complete septate uterus with OHVIRA in the English literature. Haddad et al^3^ in the year 1999 reported septate uterus with obstructed hemivagina in 9 out of 42 patients having blind hemivagina. Shavell et al^4^ in 2009 reported OHVIRA with complete septate uterus in a 19-year-old female. Fedele et al^5^ could find only 10 cases of septate uterus with OHVIRA in their retrospective study of 87 patients with obstructed hemivagina. Their retrospective study spanned over a period of 30 years, emphasizing the rarity of the disease. They studied 87 patients with double uterus with OHVIRA and found that classic HWW syndrome, that is, uterus didelphys with OHVIRA, was seen in 63 cases (72.4%), 10 out of 87 patients (11.5%) had a septate uterus whereas bicornuate uterus was seen in 9 patients (10.3%). Kim et al^6^ reported pregnancy in a septate uterus with OHVIRA in 2014.

Patients with OHVIRA usually present soon after menarche with dysmenorrhoea, pelvic pain or pelvic mass. Rarely, a patient may present in early adulthood with infertility. No such complaints were reported by our patient except for mild dysmenorrhoea. She cannot be labelled as a case of primary infertility as she had been married for the last 8 months only. OHVIRA was incidentally detected while being investigated for RIF pain caused by acute appendicitis. Moreover, she did not have haematometra, haematosalpinx or endometriosis in spite of her late presentation. This might be owing to a partial decompression of haematocolpos into the left hemivagina *via* a small communication between the two hemivaginas. Moshiri et al^7^ also reported a case of HWW syndrome with a partially obstructed hemivagina.

She also had a block vertebra involving the C6–C7 vertebrae. Vertebral anomalies are found in 29% patients with MDA, which include block vertebra, spina bifida or wedged vertebral body.^8^

Our patient had an unusual variant of HWW syndrome, having a complete septate uterus with OHVIRA with block vertebra involving the C6–C7 vertebrae.

## Treatment and outcome

The patient developed an appendicular lump during her stay in the hospital, so she was managed conservatively with intravenous antibiotics and analgesics. Excision of the vaginal septum with drainage of approximately 400 cc of deep brown-coloured haemorrhagic fluid was performed 4 weeks later. Her post-operative period was uneventful.

## Learning points

1. Early diagnosis of MDA is important as it is associated with an increased risk of endometriosis, infertility and abortions.
2. MRI is the imaging investigation of choice for the diagnosis and detailed evaluation of the type and complications of MDA.
3. HWW syndrome is an unusual and special type of MDA, characterized by the presentation of uterus didelphys with OHVIRA.
4. Compete septate uterus with OHVIRA is an exceedingly unusual variant of HWW syndrome. Very few cases have been reported in the literature to date.
